# High-Speed Holographic Shape and Full-Field Displacement Measurements of the Tympanic Membrane in Normal and Experimentally Simulated Pathological Ears

**DOI:** 10.3390/app9142809

**Published:** 2019-07-13

**Authors:** Haimi Tang, Payam Razavi, Koohyar Pooladvand, Pavel Psota, Nima Maftoon, John J. Rosowski, Cosme Furlong, Jeffrey T. Cheng

**Affiliations:** 1 Center for Holographic Studies and Laser Micro-mechaTronics (CHSLT), Worcester, MA 01609, USA; 2 Mechanical Engineering Department, Worcester Polytechnic Institute, Worcester, MA 01609, USA; 3 Faculty of Mechatronics, Informatics and Interdisciplinary Studies, Technical University of Liberec, Liberec 46117, Czech Republic; 4 Systems Design Engineering Department, University of Waterloo, Waterloo, ON N2L 3G1, Canada; 5 Eaton-Peabody Laboratory, Massachusetts Eye and Ear Infirmary, Boston, MA 02114, USA; 6 Department of Otolaryngology-Head and Neck Surgery, Harvard Medical School, Boston, MA 02114, USA

**Keywords:** frequency transfer functions, high-speed digital holography, human tympanic membrane, middle-ear pathologies

## Abstract

To improve the understanding of the middle-ear hearing mechanism and assist in the diagnosis of middle-ear diseases, we are developing a high-speed digital holographic (HDH) system to measure the shape and acoustically-induced transient displacements of the tympanic membrane (TM). In this paper, we performed measurements on cadaveric human ears with simulated common middle-ear pathologies. The frequency response function (FRF) of the normalized displacement by the stimulus (sound pressure) at each measured pixel point of the entire TM surface was calculated and the complex modal indicator function (CMIF) of the middle-ear system based on FRFs of the entire TM surface motions was used to differentiate different middle-ear pathologies. We also observed changes in the TM shape and the surface motion pattern before and after various middle-ear manipulations. The observations of distinguishable TM shapes and motion patterns in both time and frequency domains between normal and experimentally simulated pathological ears support the development of a quantitative clinical holography-based apparatus for diagnosing middle-ear pathologies.

## 1. Introduction

The human middle ear, including the eardrum or the tympanic membrane (TM), transmits sound energy from the environment to the inner ear for hearing [1,2]. The mechanism of this energy transmission through the middle ear is intrinsically multifaceted and is affected by the TM’s complex geometry (shape and thickness), spatially varied mechanical properties and microstructure (radial and circumferential fibers), as well as the load from the ossicular chain and inner ear [3–15]. Middle-ear pathologies such as TM perforation, otitis media with effusion, otosclerosis, and ossicular discontinuity can result in mild to severe conductive hearing loss. Currently, in otology and audiology clinics, there is no perfect objective diagnostic tool to differentiate middle-ear diseases, and the gold standard for the diagnosis of ossicular disorders is surgical exploration with an elevation of the eardrum to visualize the ossicular chain.

Over the past decade, various non-invasive optical methods, for example, holographic interferometric methods, were developed to quantify TM dynamics such as excitation-induced displacements, and physical characteristics such as shape and thickness [11,16–26]. However, a study on the live subject remains challenging due to the natural noise, such as motions due to respiration, heartbeat, muscle tremor, etc. and high sensitivity of the optical tools. Researchers utilized a high-speed digital holographic method to minimize these undesirable effects and made quantitative measurements on live subjects possible [24,27–30].

The high-speed digital holographic system we developed measures the TM’s shape and transient acoustic-induced displacement nearly instantaneously (<200 ms) with a spatial shape resolution of 50–200 μm and a displacement resolution of 15 nm at an acquisition rate of 67,200 Hz [24,28,30–32]. This high-speed system takes into account the holographic-sensitivity vector variations due to the sample’s shape and optical configuration to derive the true surface normal motion of the TM.

To investigate if such a high-speed digital holographic (HDH) system can be used as a diagnostic tool for middle-ear pathologies, this paper describes a study on the shape and displacement measurements on post-mortem temporal bones with different experimentally introduced middle-ear pathologies. We compared the full-field-of-view shape measurements and the time and frequency domain analysis of displacements under various middle-ear conditions (open and closed middle-ear cavity, two levels of fluid injection into the middle-ear cavity, stapes immobilization, and incudo-stapedial (IS) joint interruption) to identify TM motion patterns, and data trends associated with each middle-ear condition.

## 2. Materials and Methods

### 2.1. Principle of High-Speed Holographic for Shape and Displacement Measurements

#### 2.1.1. Multiple Wavelength Holographic Interferometry (MWHI) Method for Shape Measurement

The multiple wavelength holographic interferometry (MWHI) shape measurements are based on the principle of variations in the wavelength of illumination with a constant optical path length (OPL)(i.e., a stationary sample) [33–37]. The relation between the interference phase Δγ, the constant OPL, and wavelengths λ_1_ and λ _2_ at two hologram exposures is described as follows:

(1) $$\Delta \gamma =\frac{2\pi }{\lambda_{1}}OPL-\frac{2\pi }{\lambda_{2}}OPL=\frac{2\pi }{\Lambda }OPL,$$

where Λ is the synthetic wavelength:

(2) $$\Lambda =\frac{\lambda_{1}\lambda_{2}}{\lambda_{2}-\lambda_{1}}.$$

The synthetic wavelength Λ defines the sensitivity of the measurement. Assuming several synthetic wavelengths, the longest one determines the unambiguous measurement range, while the shortest synthetic wavelength gives the lowest uncertainty.

As described in [31], the MWHI method has been designed to sweep the synthetic wavelength allowing multiple measurements of shape in a short time. A tunable laser operating in open loop mode is used as a coherent light source, and continual wavelength tuning is achieved by changing the laser cavity length while keeping the number of waves in the cavity constant [38]. In this paper, a temporal unwrapping approach is applied to ensure that the measurement always yields the maximum resolution of a given wavelength tuning without spatial unwrapping errors.

During the wavelength tuning, N phase-shifted high-speed digital holograms are captured. The Pearson correlation of a 3 by 3 pixel kernel of the first image in the high-speed array and the rest are calculated, which gives information about optical phase change Δ*ϕ* [32] as follows:

(3) $$\rho \left(I_{1}(\text{x},\text{y}),I_{n}(\text{x},\text{y})\right)=\frac{{\left(r+1\right)}^{2}+4r\cos(\Delta \phi )}{{\left(r+1\right)}^{2}+4r},$$

where *ρ*( ) is the Pearson correlation operator, *I*_1_ is the first hologram, *I*_*n*_ is the *n*th holograms, and *r* is the object and reference beam ratio *r* = *I*_*o*_/*I*_*r*_.

Holograms with phase change Δ*ϕ* of π/2,π, and 3π/2 are identified from the image arrays to calculate the optical phase *F* of the measurement at each wavelength using the 4 step phase shifting equation [39]:

(4) $$F=\text{arctan}\frac{I_{3\pi /2}-I_{\pi /2}}{I_{1}-I_{\pi }},$$

where *I*_π_, *I*_π/2_, and *I*_3π/2_ are the π/2, π, and 3 π/2 phase shifted holograms.

The optical phase difference Δ*F*_*N*−1_ of two consecutive high-speed phase measurements are calculated, and 2D Goldstein’s branch cut method [40] is applied to remove any phase jumps. The differential approach provides measurements with lower dynamic ranges, and therefore, the spatial unwrapping is more reliable. Moreover, if the number of phase sampling *N* is high enough, the optical phase difference has no 2π phase jumps, so the spatial unwrapping can be completely avoided.

The total phase change Δγ of the entire wavelength tuning, ensuring the lowest uncertainty, is

(5) $$\Delta ϒ=\sum_{2}^{N}\Delta \Phi_{k}.$$

Assuming perpendicular illumination and observation of the object, the surface *z* component of a point (*x, y*) on the sample is

(6) $$z(x,y)=\frac{\Lambda }{4\pi }\Delta ϒ(\text{x},\text{y}).$$

#### 2.1.2. High-Speed Digital Holographic (HDH) Method for Displacement Measurement

We have previously developed and implemented the HDH method based on correlation interferometry that could “instantaneously” measure the full-field-of-view (>200,000 points at 67,200 camera frame rate) transient displacements of the TM in response to impulsive acoustical and mechanical excitations [24,28,30]. As described in [31], the HDH uses Pearson’s correlation to locate two reference frames *I*_*ref*_ and *I*_*ref*_ +π/2 with 0 and π/2 prior to the sample’s excitation based on Equation (3). The zero-order terms of all correlated kernels are removed by summation of reference frames that are within one period of optical phase shifts. The interference phase Δ*θ* as a result of the sample’s motion of a small window centered at (*x, y*) is

(7) $$\Delta \theta (t)=\text{arctan}\frac{\rho \left(I_{\text{ref}},I_{\text{def}}(t)\right)}{\rho \left(I_{\text{ref}+\pi /2},I_{\text{def}}(t)\right)}.$$

Studies of in-plane and out-of-plane motions of the human tympanic membrane [41] confirm that TM has a thin-shell structure with the dominant displacement component along the surface normal. Using the information of the shape and the geometry of the experiment setup, the surface normal (***n***) of the TM surface and the sensitivity vectors (***K***) [28] of the holographic measurement are computed at each point of the TM surface. The magnitude of the displacement *s*(*t*), therefore, can be expressed as

(8) $$s(x,y,t)=\frac{\Delta \theta (x,y,t)}{K(x,y)\Delta n(x,y)}.$$

### 2.2. Experimental Setup and Procedures

**Figure 1.**
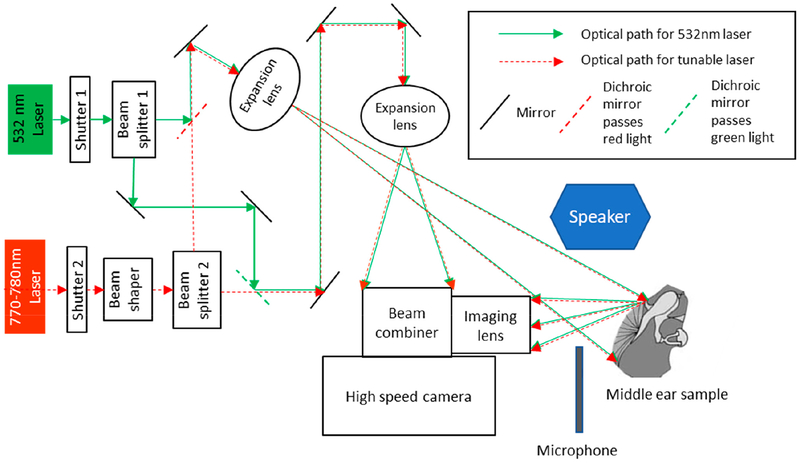
Schematic of high-speed digital holographic (HDH) system [4]. Schematic of high-speed digital holographic (HDH) system [4]. The 532 nm laser is used for displacement measurements, and the 770–780 nm tunable laser is used for shape measurements. The two lasers are coupled into the same optical path two lasers are coupled into the same optical path to ensure the shape and displacement measurements are in the same coordinate system avoiding the need for image registration.

The schematic diagram of the HDH system used in this study is shown in Figure 1. The details of the HDH system and acoustic measurement setups can be found in our previous publication [31]. Three fresh non-fixed human postmortem temporal bones were used for the measurements. The cartilaginous and bony parts of the ear canal were removed to provide optical access to the TM. The middle-ear cavity was opened to check the health of the middle-ear ossicles, and then it was sealed using the sealing cement. A small metal tube was installed through the eustachian tube for injecting fluid into the sealing cement. A small metal tube was installed through the eustachian tube for injecting fluid into the middle-ear cavity. The TM surface was sprayed with a thin layer of HOLBEIN water soluble oil color (commercial trade name: TITANIUM WHITE) using a commercially available airbrush (MASTER® Model S68).

**Figure 2.**
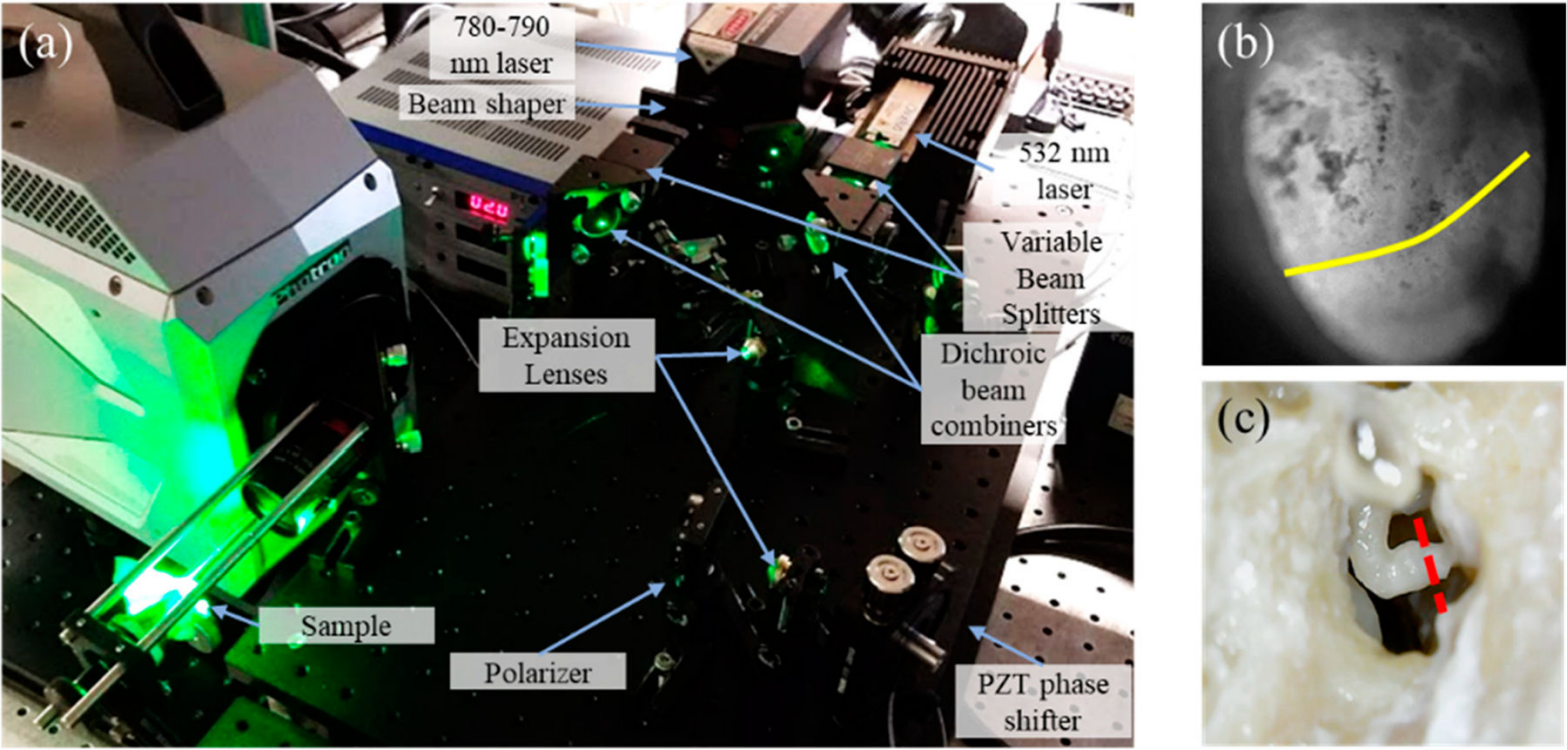
(**a**) Shows the setup, measuring instrumentations, and the sample; and (**b**) is a photograph of the human postmortem tympanic membrane (TM) with half level saline injected. The yellow contour shows the level of fluid. (**c**) Shows the incudo-stapedial (IS) joint. The red dash shows where the IS joint is interrupted.

Primary experiments measured the transient TM responses to a short acoustic click (i.e., a 50 μs square pulse) generated by a speaker, as shown in Figure 1, and the TM shape information to calculate the true out-of-plane motion of the entire TM [4]. We also acquired the acoustic excitation profile using a high-frequency microphone situated close to the TM, as shown in Figure 1. After the control measurement on the normal ear (closed middle-ear cavity), the sample was subjected to a series of manipulations that simulated different middle-ear conditions and pathologies. The manipulations included: (i) open middle-ear cavity, (ii) closed middle-ear cavity, (iii) injecting saline into the middle-ear cavity to the level of half TM in contact with fluid, as shown in Figure 2b, (iv) injecting saline until the full TM was in contact with fluid, (v) removing the injected fluid, (vi) immobilizing the footplate of the stapes with super glue, and (vii) interrupting the incudo-stapedial (IS) joint, as shown in Figure 2c. The shape and transient acoustic-induced displacements were measured at each manipulation (experimental setup is shown in Figure 2a). The human temporal bone study was approved by the Institutional Review Board of the Massachusetts Eye and Ear Infirmary. The use of de-identified human cadaveric tissue (only age and gender information are available) does not require a human subject study protocol.

### 2.3. Experimental Modal Analysis

Frequency response function (FRF) matrices computed from the ratio of the output displacements s and the input sound pressure *p* in the frequency domain were calculated for all the TM surface points with various middle-ear manipulations.

(9) $$\text{FRF}(x,y,\omega )=\frac{FFT(s(x,y,t))}{FFT(p(t))},$$

where *x, y* are the coordinates of TM points, *t* is time, and ω is the frequency.

FRF(*x, y, ω*) was then flattened into a 2D matrix *H*(*n*,ω) where *n* is the number of the nodes. FFT denotes fast Fourier transform.

Complex mode indicator functions (CMIFs) were calculated by taking singular value decomposition ([17], Equation (1)) of each FRF matrix,

(10) $$[H]=[U][\Sigma ]{\left[V\right]}^{H},$$

where [*H*] is the flattened frequency response function matrix; [*U*] is the left singular vector matrix (unitary); [Σ] is the singular value matrix (diagonal); [*V*] is the right singular vector matrix (unitary); and []^*H*^ marks the Hermitian (conjugate transpose) of matrix. The CMIF is defined as the main diagonal elements of the singular value matrix.

(11) CMIF=diag[Σ]

## 3. Results

### 3.1. Representative Shape Measurement Results

**Figure 3.**
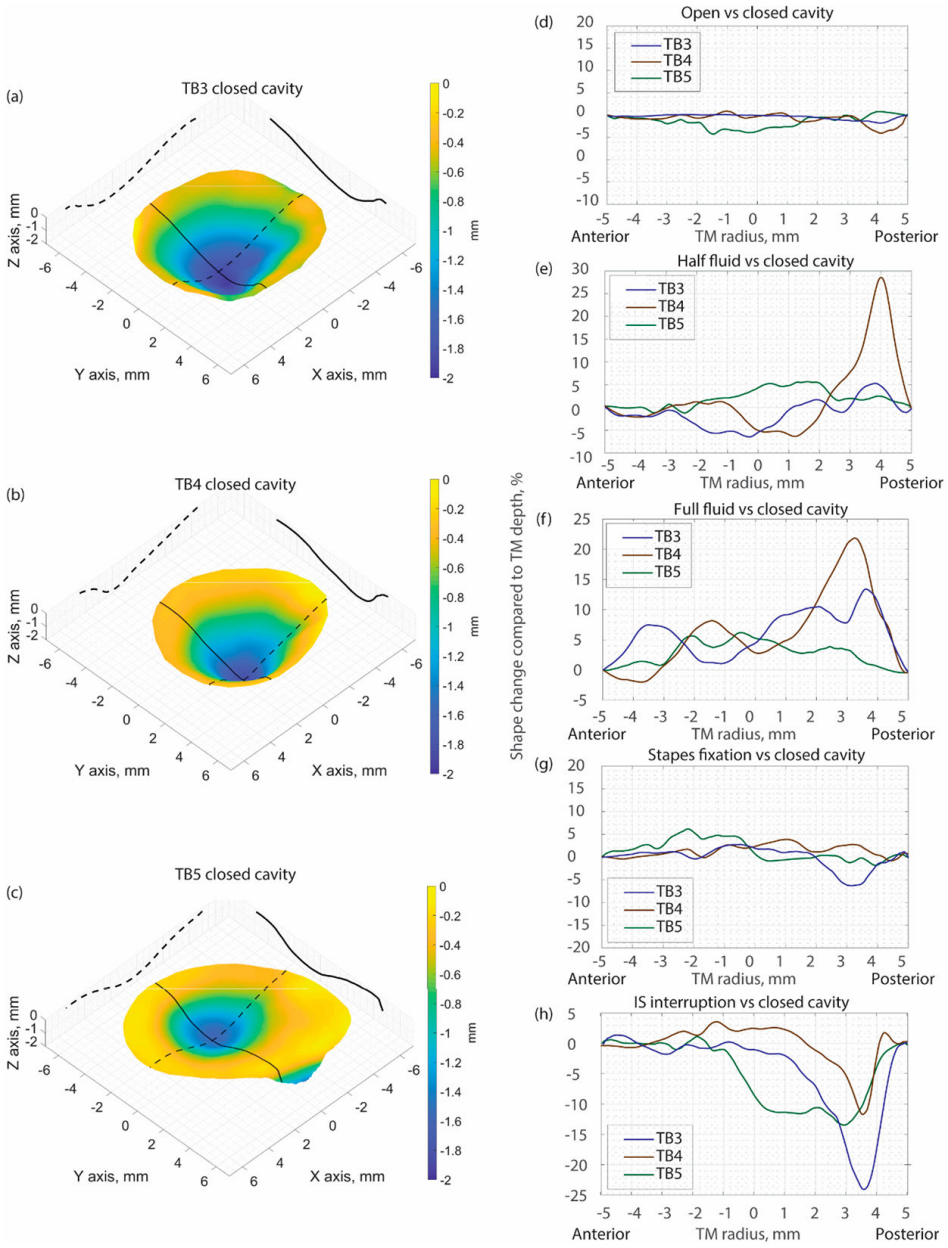
(**a**):TB3 shape when the middle-ear cavity is closed; (**b**):TB4 shape when the middle-ear cavity is closed;(**c**): TB5 shape when the middle-ear cavity is closed; Comparison of TM shape along the black solid line marked in the left panel in TB 3~5 (**d**): between open and closed cavity condition; (**e**): between half fluid injection and closed cavity condition; (**f**): between full fluid injection and closed cavity condition; (**g**): between Stapes fixation and closed cavity condition; (**h**): between IS point interruption and closed cavity condition.3.2. Representative Displacement Measurement Results

The MWHI method has been verified to reach an accuracy of 0.045 mm and exhibited repeatable measurements with 17 μm accuracy on a (National Institute of Standards and Technology) traceable ball and artificial samples [31]. This paper presents the results of TM shape measurements from three post-mortem human temporal bones (TB3, TB4, and TB5) under different manipulations. The 3D shapes of the TM of the three samples measured with a closed middle-ear cavity are shown in Figure 3a–c with laser continuous wavelength tuning from 779 to 779.4 nm (i.e., maximum synthetic wavelength 1.7 mm), while N (N = 17) of phase samplings are performed to capture the optical phase at different wavelengths during wavelength tuning. The depth of the TM is rendered by color as shown in the color bar, where the TM annulus is set as zero (yellow), and the value increases negatively towards the middle-ear cavity side. In Figure 3d–h, the difference of the TM shape along the solid black line across the TM surface, as shown in Figure 3a, between the closed cavity (as reference) and different manipulations are shown. The comparison between the open cavity and the closed cavity shows no significant TM shape change in all three TBs (Figure 3d). The fluid injection induces more significant TM shape change where the fluid in contact with the TM pushes the TM surface outwards towards the ear canal side, yielding positive TM shape change values as shown in Figure 3e,f. Note in Figure 3e only half of the TM (to the right side of the plot) is in contact with the fluid. Stapes immobilization did not introduce significant TM shape change, as shown in Figure 3g. The IS joint interruption induced more significant changes to the TM shape which generally occurred at the posterior part of the TM, and the direction of the shape change was towards the middle-ear cavity (as shown in negative values in Figure 3h).

**Figure 4.**
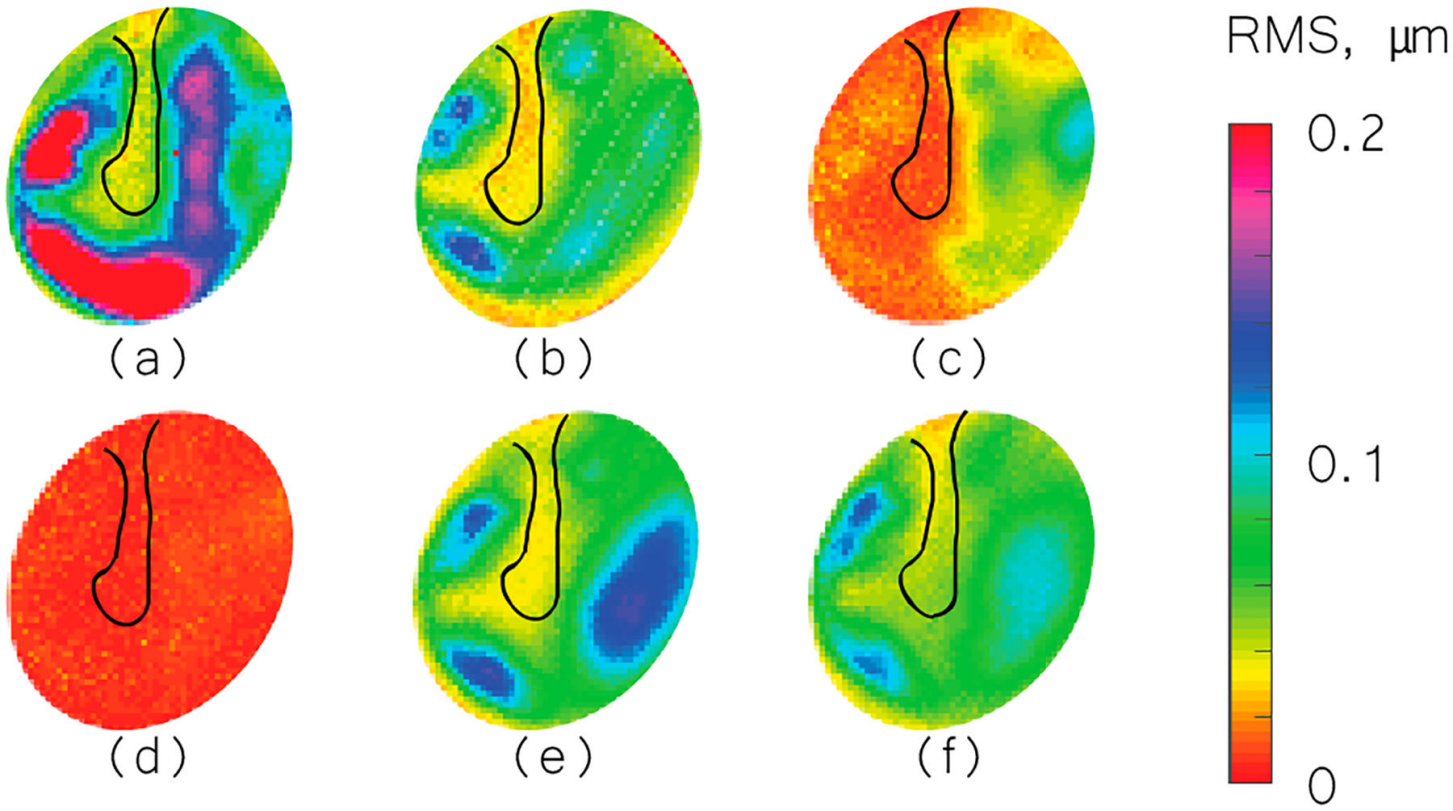
Root mean square (RMS) of TB3 displacement under different conditions. (**a**) Open cavity; (**b**) closed cavity; (**c**) half fluid in contact; (**d**) full fluid in contact; (**e**) stapes immobilization; and (**f**) IS joint interruption.

**Figure 5.**
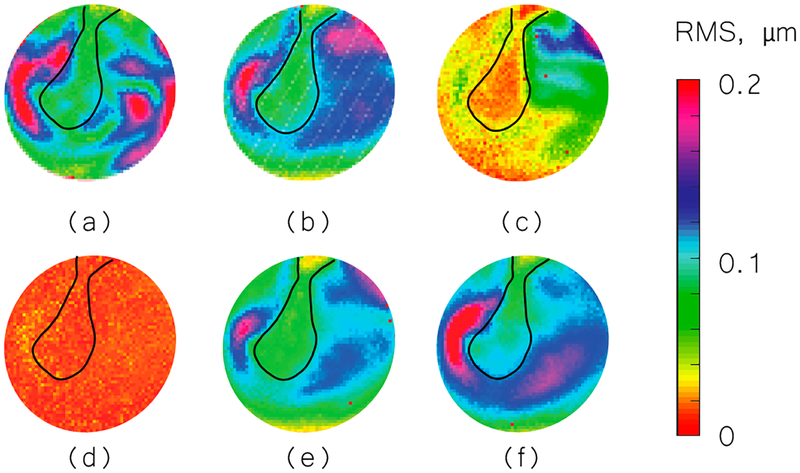
RMS of TB4 displacement under different conditions. (**a**) Open cavity; (**b**) closed cavity; (**c**) half fluid in contact; (**d**) full fluid in contact; (**e**) stapes immobilization and (**f**) IS joint interruption.

**Figure 6.**
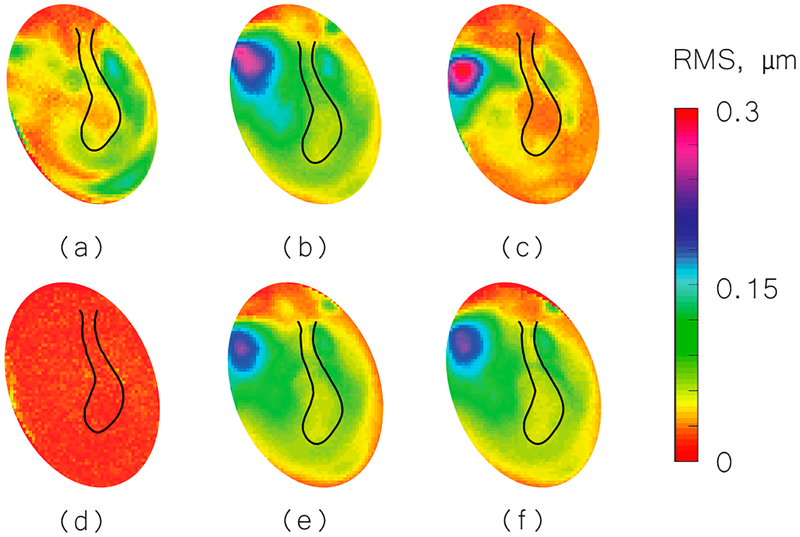
RMS of TB5 displacement under different conditions. (**a**) Open cavity; (**b**) closed cavity; (**c**) half fluid in contact; (**d**) full fluid in contact; (**e**) stapes immobilization; and (**f**) IS joint interruption.

**Figure 7.**
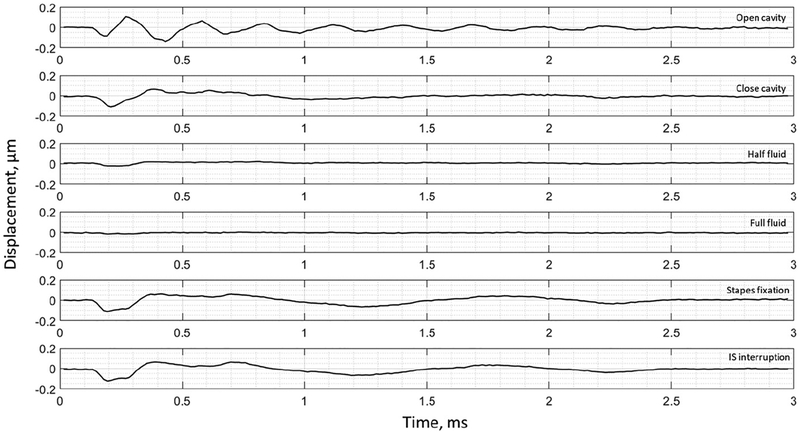
Umbo displacement of TB3 under different cases (umbo location is marked as the intersection of the solid black and dashed black lines in Figure 3a).

**Figure 8.**
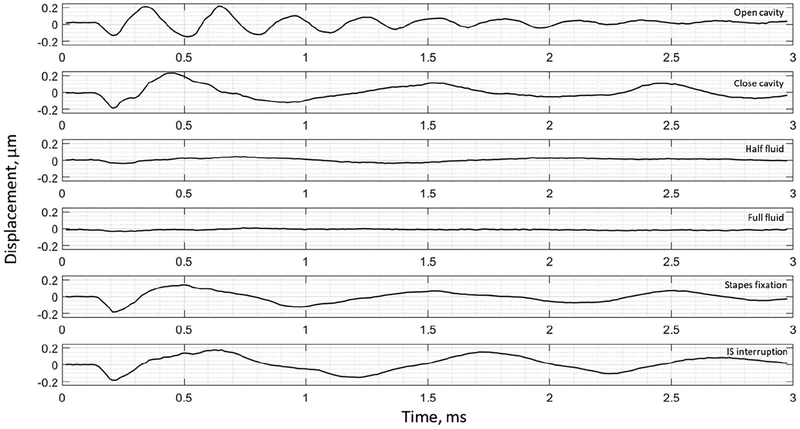
Umbo displacement of TB4 under different cases (umbo location is marked as the intersection of the solid black and dashed black lines in Figure 3b).

**Figure 9.**
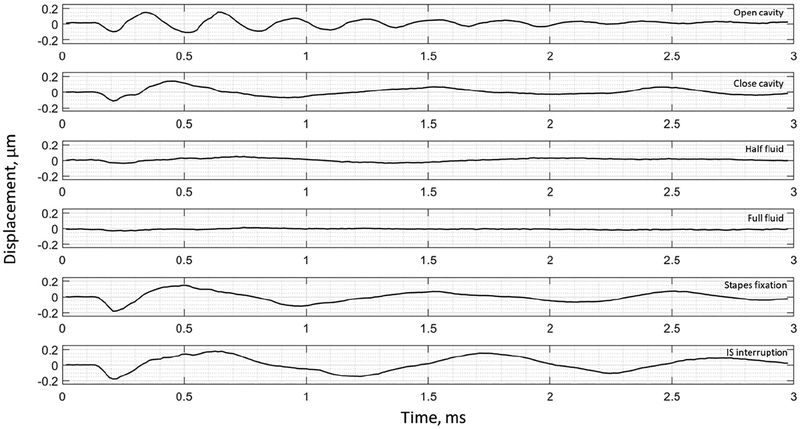
Umbo displacement of TB5 under different cases (umbo location is marked as the intersection of the solid b lack and da shed black lines in Figure 3c).

We used the shape information to derive TM transient surface-normal displacements. We investigated using the root mean square (RMS) of the motion of the entire TM surface to define an indicator to evaluate and distinguish different middle-ear manipulations. We also compared the umbo displacement in the time domain among different middle-ear manipulations. Figures 4–6 show the RMS of TM surface motions over the first 3 ms after the TM receives the acoustic excitation for three TBs—under different middle-ear conditions. Figures 7–9 show the umbo motion during the same time period under different manipulations for three bones. From the RMS results, we observed that an open cavity case always has a more complex distribution of the RMS than a closed cavity case. For half fluid injection cases, only the area not in contact with fluid shows significant RMS values of motion (right side of the TM in Figures 4c and 5c and left side of the TM in Figure 6c, the difference is caused by the different sides of the ear these specimens come from (TB3: 75 year old female, left ear; TB4: 61 year old male, left ear; TB5: 76 year old female, right ear)). When the middle-ear cavity is fully filled with fluid, the RMS value of TM motions is almost zero. Stapes immobilization and IS joint injection exhibit RMS motion patterns that are similar to those observed in the normal case.

The umbo displacement profiles with time show distinguishable differences among different middle-ear conditions for all three bones, as shown in Figures 7–9. In an open cavity case, the umbo undergoes more periods of motion compared to a closed cavity case, or umbo motion damps out faster in the closed cavity case. Half-fluid and full-fluid injections suppress umbo motion to very little or no sensible motion. The stapes immobilization and IS joint interruption alter the umbo motion in an analogous fashion with little distinguishable differences between the two cases.

**Figure 10.**
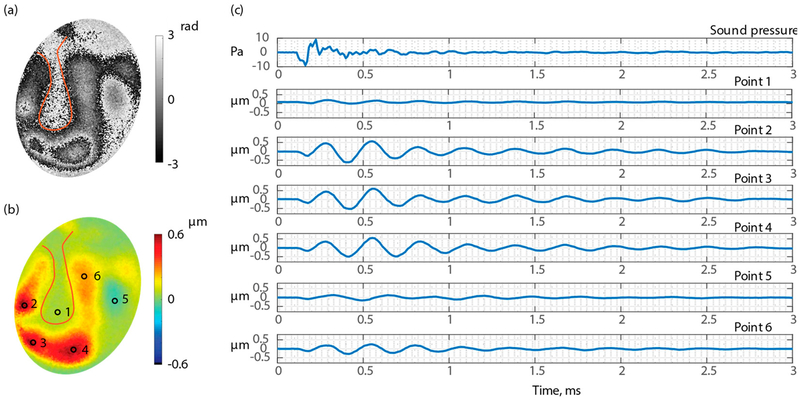
Waveforms of six points of the surface of the TM of TB3 when the middle-ear cavity is open. (**a**)Wrapped optical phase 0.56 ms after excitation; (**b**) TM surface normal displacement 0.56 ms after excitation; and (**c**) time waveforms of six points marked in part (**c**).

Figure 10 shows the displacement of TB3 under an open middle-ear cavity. Figure 10a shows the measured optical phase at an arbitrary time, Figure 10b shows the unwrapped and scaled surface’s normal displacement calculated from Figure 10a, and Figure 10c is the time waveform of the acoustic excitation signal (first row) and displacements of six discrete points marked in Figure 10b. The results show that different regions of the TM respond to the excitation differently, and that different regions of the TM reach displacement maximum and minimum at different times, and eventually settling at different times. The time of delay for each point to start the motion compared to the acoustic signal is also different point by point, suggesting that there are traveling waves forming on the surface of TM.

### 3.2. Frequency Analysis: Complex Mode Indicator Functions

**Figure 11.**
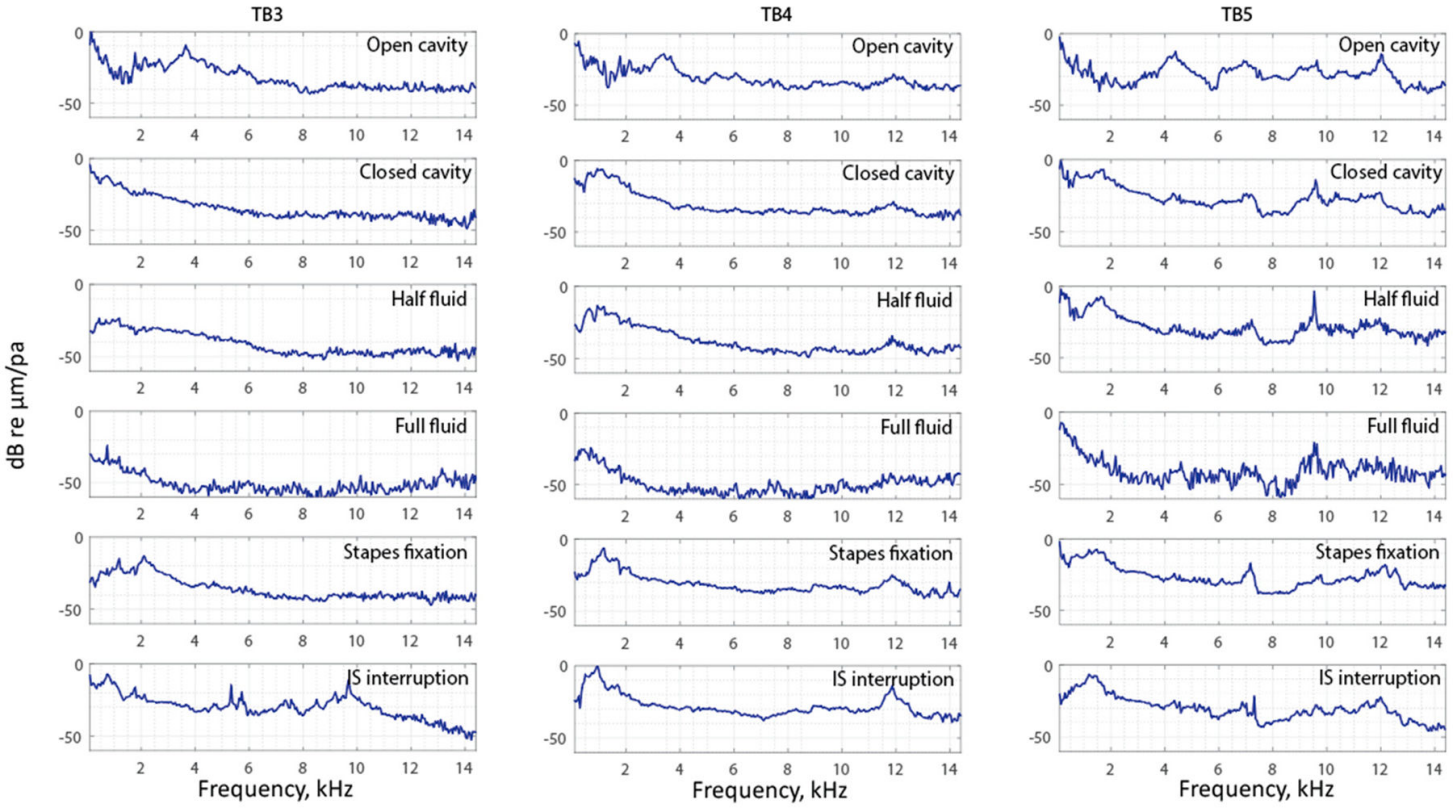
The complex model indicator function of TB3–5 under different conditions.

**Figure 12.**
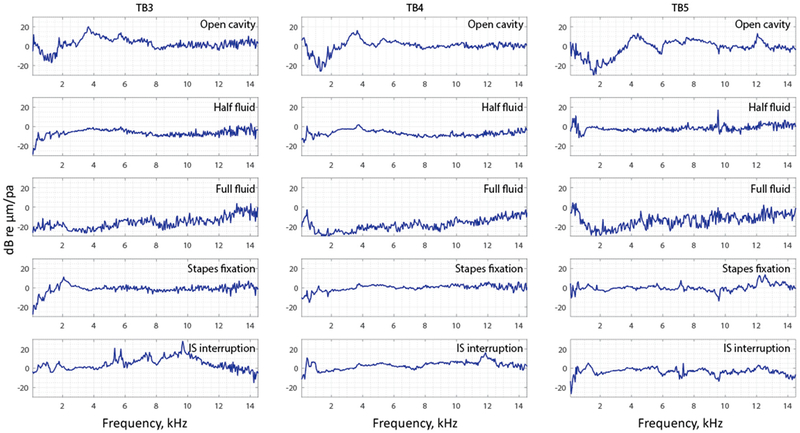
The complex model indicator function subtracted by the closed cavity case of TB3–5.

To visualize and compare all the data of the entire surface of the TM across different manipulations, the complex mode indicator function (CMIF) was used as a generalized presentation of the membrane’s frequency response. Figure 11 shows CMIFs computed from all three temporal bones with different middle-ear manipulations. The horizontal axis is the frequency in kHz (kilohertz), and the vertical axis is the singular value in decibels, which are the square root of the eigenvalues of the frequency response function (FRF) matrix. We observed that CMIF changes with different middle-ear conditions. We defined the closed cavity condition, as shown in the second row of Figure 11, as the baseline and subtracted other obtained CMIF data from this baseline for further comparison in Figure 12. The open cavity condition generally decreases CMIF amplitude at low frequencies around 1 kHz and induces a major peak at 3 to 5 kHz in all three TBs. A half fluid condition in TB3 and TB4 shows lower CMIF amplitude across the entire frequency spectrum compared to the baseline closed cavity case, although the decrease of CMIF in TB5 is relatively small. Also, for the full fluid case, all three samples have the lowest CMIF amplitudes, consistent with TM motions that are significantly reduced by fluid inside the middle-ear cavity. The CMIFs under the stapes immobilization condition at low-frequency ranges(0.5–2.5 kHz) for TB3 and TB4 are significantly reduced. However, this reduction is not obvious in TB5. Finally, the IS joint interruption condition introduces several additional peaks for CMIFs, particularly at mid- and high-frequency ranges.

## 4. Discussion

This study provides a complete description of TM mechanics, including the 3D shape information and full-field TM surface normal vibration in both the time and frequency domains.

The shape results show that the TM shape is affected by various middle-ear manipulations. The different manipulations overall introduce a maximum shape change of less than 0.3 mm. The fluid injection pushes the TM to deform towards the ear canal side, while the IS joint interruption seems to pull the TM toward the middle-ear cavity side, which is interesting and may suggest the cochlear load produced tension on the TM. No differentiable shape change is identified for open cavity and stapes immobilization.

Displacements in the time domain show we can confidently distinguish half fluid injection and full fluid injection by checking the RMS results and the open cavity case. Fluid injections will surpass the TM motion. Opening the cavity causes the TM surface to displace more compared to the normal middle-ear cavity condition (closed cavity).

In the frequency domain, we observed trends in different CMIFs associated with different middle-ear conditions. In open cavity cases, the first significant CMIF peak appears around 4 kHz(3.7 kHz for TB3, 3.5 kHz for TB4, and 4.3 kHz for TB5). Half fluid and full fluid injections decrease the CMIF across the whole frequency range. Stapes immobilization reduces the CMIF at a lower frequency up to 2.5 kHz for TB3 and TB4 and increases it for TB5 at a higher frequency (12–14 kHz). IS joint interruption adds additional peaks to the CMIF at different frequencies.

Time domain and frequency domain analysis results can separate different middle-ear pathologies, suggesting the potential for clinical diagnosis. In the future work, we will investigate the accuracy of potential diagnostics through further experiments—identify trends in the data associated with different pathologies and test the sensitivity and selectivity of these analyses for clinical diagnosis. Artificial intelligence (AI) and data mining will be applied to automate the analysis process and assist in the separation of normal and diseased states.
